# Halo score (temporal artery, its branches and axillary artery) as a diagnostic, prognostic and disease monitoring tool for Giant Cell Arteritis (GCA)

**DOI:** 10.1186/s41927-020-00136-5

**Published:** 2020-08-18

**Authors:** Alwin Sebastian, Kornelis S. M. van der Geest, Fiona Coath, Prisca Gondo, Abdul Kayani, Craig Mackerness, Bernard Hadebe, Sue Innes, Jo Jackson, Bhaskar Dasgupta

**Affiliations:** 1 Rheumatology, Mid and South Essex University Hospital Groups, Southend University Hospital, Westcliff-On-Sea, Essex, UK; 2 University of Essex, Colchester, UK; 3 Rheumatology and Clinical Immunology, University Medical Center Groningen, University of Groningen, Groningen, The Netherlands; 4 Norfolk and Norwich University hospitals NHS Foundation Trust, Colney Ln, Norwich, UK; 5 R&D, Mid and South Essex University Hospital Groups, Southend University Hospital, Westcliff-On-Sea, Essex, UK; 6 School of Sport, Rehabilitation and exercise sciences, Colchester campus, University of Essex, Colchester, UK

**Keywords:** Outcomes in GCA, Risk stratification, Prognostic factors, Halo score, GCA probability score, Clinical severity index, Glucocorticoid toxicity

## Abstract

**Background:**

Giant cell arteritis (GCA) is a common large vessel vasculitis of the elderly, often associated with sight loss. Glucocorticoids (GC remain the mainstay of treatment, although biologic treatments have been approved. Biomarkers predicting disease severity, relapse rates and damage are lacking in GCA.

EULAR recommends ultrasound (US) as the first investigation for suspected GCA. The cardinal US finding, a non-compressible halo, is currently categorised as either negative or positive. However, the extent and severity of this finding may vary.

In this study, we hypothesise whether the extent and severity of the halo sign [calculated as a single composite Halo score (HS)] of temporal and axillary arteries may be of diagnostic, prognostic and monitoring importance; whether baseline HS is linked to disease outcomes, relapses and damage; whether HS can stratify GCA patients for individual treatment needs; whether HS can function as an objective monitoring tool during follow up.

**Methods:**

This is a prospective, observational study. Suspected GCA Participants will be selected from the GCA FTC at the participating centres in the UK. Informed consent will be obtained, and patients managed as part of standard care. Patients with GCA will have HS (temporal and axillary arteries) measured at baseline and months 1,3,6 and 12 long with routine clinical assessments, blood sampling and patient-reported outcomes (EQ5D). Non-GCA patients will be discharged back to the referral team and will have a telephone interview in 6 months.

We aim to recruit 272 suspected GCA referrals which should yield 68 patients (25% of referrals) with confirmed GCA. The recruitment will be completed in 1 year with an estimated total study period of 24 months.

**Discussion:**

The identification of prognostic factors in GCA is both timely and needed. A prognostic marker, such as the HS, could help to stratify GCA patients for an appropriate treatment regimen. Tocilizumab, an IL-6R blocking agent, switches off the acute phase response (C-Reactive Protein), making it difficult to measure the disease activity. Therefore, an independent HS, and changes in that score during treatment and follow-up, maybe a more objective measure of response compare to patient-reported symptoms and clinical assessment alone.

## Background

Giant cell arteritis (GCA) is a common form of systemic vasculitis characterised by granulomatous inflammation of large and medium-sized arteries [1]. GCA predominantly affects Caucasian, older people (> 50 years), with a peak incidence among those 70–80 years old [1, 2]. The incidence of GCA rises with increasing age, ranging from 2.6 per 100,000 in patients aged 50–59 to 44.6 per 100,000 in patients over the age of 80 [3]. GCA predominantly involves branches of the external carotid arteries such as the temporal arteries and the aorta and its large branches, including the subclavian and axillary arteries. Common presenting symptoms include new headache, scalp tenderness, jaw claudication, diplopia and amaurosis fugax [1, 2, 4]. GCA can cause significant morbidity and ischaemic complications, including irreversible sight loss. Other complications include aortitis, myocardial infarction and stroke. The 1990 American College of Rheumatology (ACR) classification criteria were not intended for diagnosis [5] and may not be accurate, particularly for cases with ophthalmic involvement [6]. The criteria have low specificity and predictive values [2, 7, 8]. Screening tests are vital as the GCA symptoms can be often non-specific and missing the diagnosis can be devastating [9].

Glucocorticoids (GC) have remained the cornerstone of treatment for GCA [10], although cohort studies show only 15–20% sustained remission with glucocorticoids alone Glucocorticoid-sparing treatments in GCA are also needed due to the harmful effects of long-term glucocorticoid use. This includes hypertension, hyperglycaemia, osteoporosis, cushingoid changes, mood disturbance and electrolyte imbalance, but this is not an exhaustive list [11, 12]. It is recommended to start GC immediately in strongly suspected GCA pending investigations [13]. Targeted treatments have recently been introduced, but heterogeneity in disease outcomes has still been observed. In GiACTA, the landmark trial of Tocilizumab in GCA that provides the evidence base for its current use, 42% of participants randomised to weekly Tocilizumab still did not achieve sustained remission [14]. Currently, validated biomarkers predicting disease severity, relapse rates and damage are lacking in GCA.

A positive temporal artery biopsy (TAB) has been the gold standard for histological diagnosis of GCA [15–17]. However, a biopsy is invasive, and it lacks sensitivity. This is particularly true in extra-cranial involvement, termed large-vessel GCA (LV-GCA), where access to sample material has obvious practical constraints and is usually identified incidentally following cardiovascular surgery [18]. Non-invasive imaging techniques, including ultrasound (US), Magnetic resonant image (MRI) and position emission tomography (PET-CT) are increasingly being used to identify these patients [19–21].

Ample evidence now indicates that US of temporal arteries can promptly diagnose cranial forms of GCA, as well as screening for LV-GCA at the axillary arteries [22]. US is a safe, non-invasive and higher sensitivity, particularly in extra-cranial disease. It is a relatively quick procedure [23], often delivered as a point of care test, well tolerated by patients and is suitable for follow-up examinations. Timely diagnosis of GCA by ultrasound in GCA fast track clinics has resulted in a significant reduction in permanent visual loss [24–26].

The EULAR recommendations for imaging in Large Vessel Vasculitis recommend US of temporal and/or axillary arteries as the first imaging modality, where there is adequate expertise and equipment, particularly in patients with suspected predominantly cranial GCA [27]. Estimation of GCA probability has become important given recent EULAR recommendations suggesting different diagnostic strategies in patients with low, intermediate or high GCA probability. In patients where there is a high clinical suspicion of GCA and an initial positive imaging test e.g. US, the diagnosis of GCA may be made without additional investigations (e.g. biopsy or further imaging). In patients with a low clinical probability and a negative imaging result, the diagnosis of GCA can be considered unlikely, and the patient reassured [18]. There is also a report from Southend suggesting that the ‘pre-test GCA Probability Score’ may be a useful tool for rating the pre-test probability of GCA, stratifying patients into ‘low’ or ‘not-low’ probability groups [28]. This score may also reflect clinical severity and extent of disease.

The main finding on US in GCA patients is the halo sign: non-compressible hypoechoic wall swelling [29, 30]. Several studies have been conducted to investigate the accuracy, construct and criterion validity of US in the diagnosis of GCA [31–34]. The latest meta-analysis of prospective studies has shown a pooled sensitivity of 77% and a pooled specificity of 96% for temporal artery US when compared to the final clinical diagnosis of GCA [35]. US allows measurement of the arterial intimal media complex (IMC). Studies show that at the age of 70 years the temporal artery has a normal IMC diameter of about 0.2 mm, whilst inflamed temporal arteries have a diameter of 0.5–0.9 mm [27, 36]. Axillary arteries of patients aged about 70 have a normal IMC diameter of 0.6 mm, whilst patients with extra-cranial GCA have an average diameter of 1.6–1.7 mm [36, 37]. A cut off value was determined at 1.0 mm [36]. Currently, the temporal artery US of GCA patients are categorised as either negative or positive. However, variation in extent and severity of these findings on temporal and axillary artery US in GCA is observed [38]. We have recently developed an ultrasonographic halo score that correlates with arterial inflammation in GCA [39]. In the current study, will further investigate the novel halo score as a diagnostic, prognostic and disease monitoring tool for GCA.

**Fig. 1:**
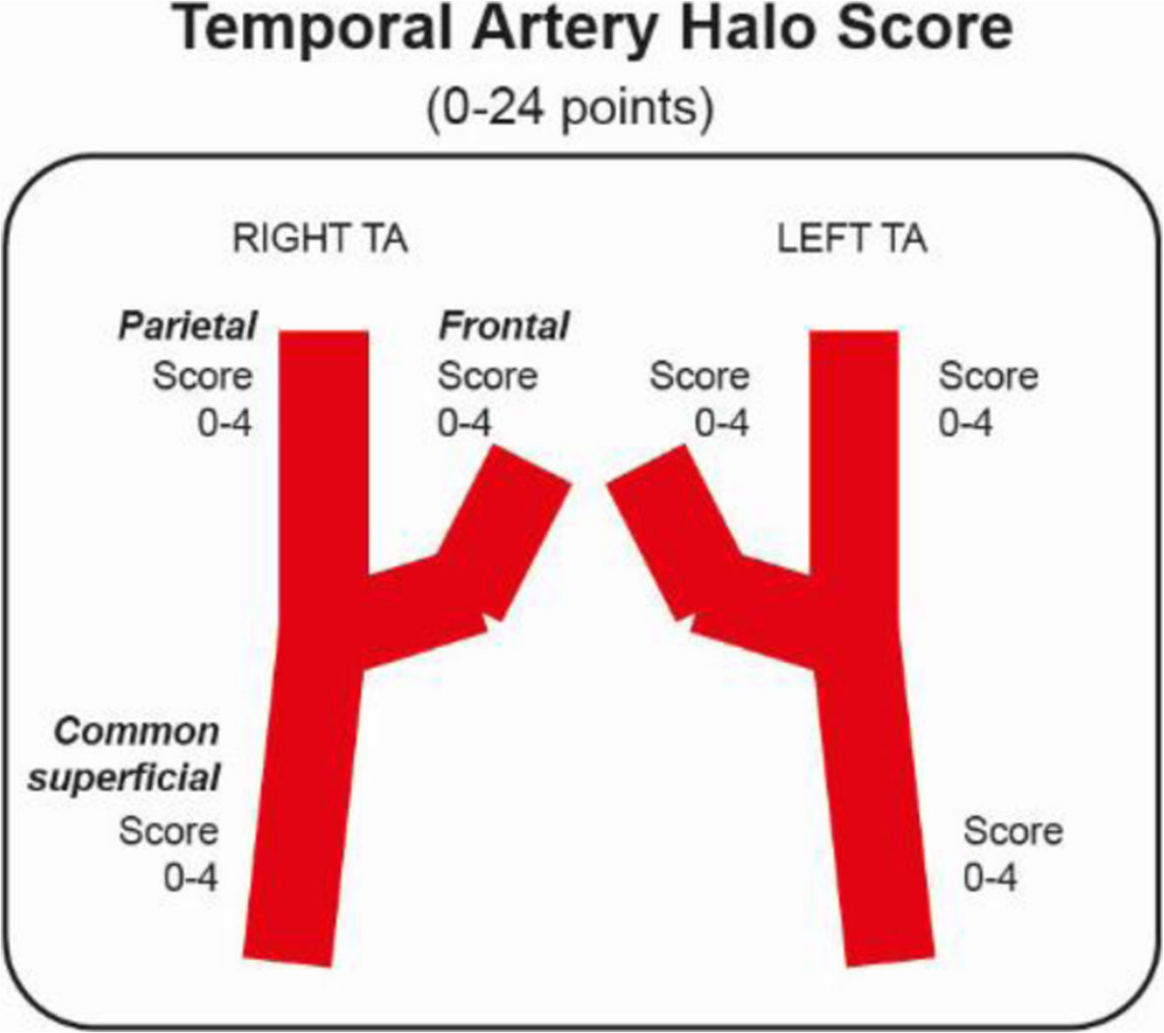
Diagram demonstrating the six temporal artery segments for calculating the temporal artery halo score

**Fig. 2:**
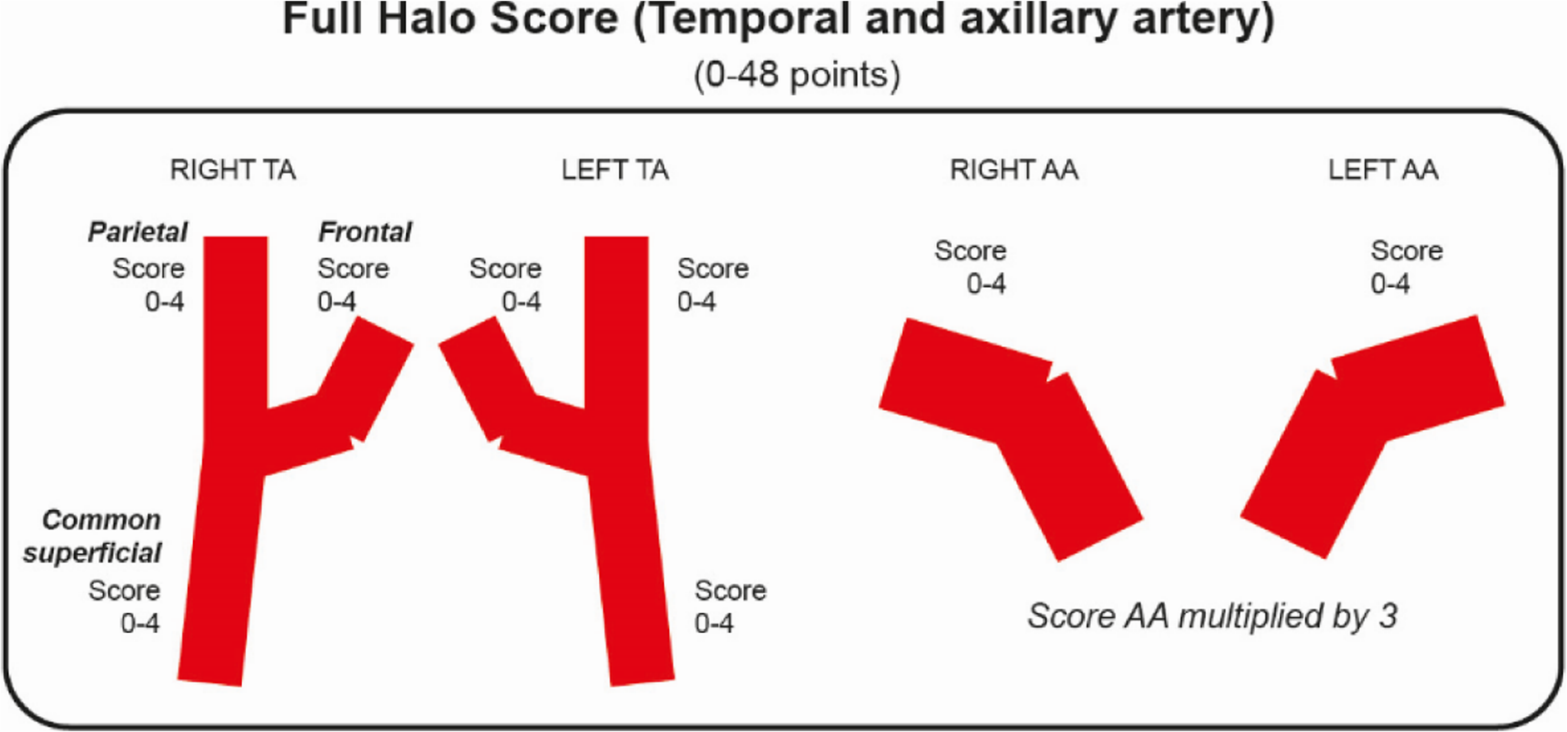
Diagram demonstrating the six temporal artery segments and two axillary arteries for calculating the total artery halo score

We will systematically measure the extent and severity of the halo sign. Bilateral US assessment of the common temporal artery, the parietal branch, the frontal branch and axillary arteries will be performed (Figs. 1 and 2). The halo sign at each branch of the common temporal, parietal and frontal arteries will be scored 0–4 points, giving a maximum possible halo score (HS) score of 24 (Table 1). At the axillary arteries, the IMT will be scored 0–4 points on each side, allowing a maximum total score 8, which will be multiplied by 3 (Fig. 2). A total halo score (THS) will be constructed by adding the scores of the temporal artery branches with the axillary artery score.

**Table 1:** Halo Score Grading

Halo Grading	Common superficial TA halo thickness (mm)	Parietal TA halo thickness (mm)	Frontal TA halo thickness (mm)	Axillary artery halo thickness (mm)
Grade 0	0.3 or less	0.2 or less	0.1 or less	0.5 or less
Grade 1	0.4	0.3	0.2	0.6
Grade 2	0.5	0.4	0.3	0.7–0.8
Grade 3	0.6–0.7	0.5*	0.4	0.9–1.5
Grade 4	0.8 or more	0.6 or more	0.5 or more	1.6 or more

Subsequently, the HS and THS will be assessed for any correlation to disease outcomes in GCA, as characterised by responsiveness to therapy - remitting, relapsing or refractory disease. Other outcome measures that may be reviewed include the development of large vessel disease and vascular damage (as assessed by cross-sectional scanning such as PET-CT), accumulation of glucocorticoid related adverse events and need for additional conventional (e.g. leflunomide, methotrexate) or biologic (Tocilizumab) DMARDs. Remitting disease in GCA is defined as a disease under sustained satisfactory control with minimum one flare during standard GC taper. The relapsing disease is where the condition initially comes under control but then flares on GC tapering. Refractory GCA patients are those who do not respond to GC at all.

The identification of prognostic factors in GCA is both timely and needed. The GiACTA trial has shown that IL-6R blocking therapy may help to sustain glucocorticoid free remission [14]. In addition, the GiACTA trial has shown that a subset of GCA patients can be quickly withdrawn from glucocorticoid therapy without the development of relapses. A prognostic marker, as outlined above, could help to stratify GCA patients to an appropriate treatment regime. IL-6R blockade switches off the inflammatory marker response, making it difficult to use traditional biomarkers such as CRP to measure disease activity. Therefore, an independent HS, and changes in that score during treatment and follow-up, maybe a more objective measure of response, rather than relying only on patient-reported symptoms and clinical assessment.

## Methods/design

### Study aims and hypothesis

To determine whether the severity of vessel wall oedema (halo/IMT) in the common temporal artery, its branches and the axillary arteries, as measured by a composite ultrasound score (THS), is of prognostic value in predicting severity and outcomes in GCA.

To determine the prognostic and monitoring value of the HS and THS in GCA, with regards to predicting outcomes (remission, refractory or relapsing disease) in GCA. We will also determine the diagnostic value of the HS and THS for discriminating GCA from non- GCA.

### Study design

This is a pragmatic, prospective, observational study.

This study will involve two specific phases

1. Initial presentation and diagnosis of GCA or non-GCA.
2. Follow-up over 12 months for GCA patients and 6 months for non-GCA patients.

#### Initial presentation and diagnosis

This phase will involve recruiting patients from the GCA FTP at participating sites. Patients recruited will be subject to inclusion/exclusion criteria detailed below. Their General Practitioner, Emergency Department or other specialities refer patients to the FTP. Patients will be invited to participate in this study by the Rheumatology Research team, who will provide them with information about the study. Patients will be informed of the phases of participation, the voluntary nature of the study, and their right to withdraw at any stage. Written consent to participate will be obtained by the researcher prior to the commencement of the screening assessments.

In this phase following will be assessed: (Additional file 1)

- Clinical history
- Clinical examination
- Routine bloods including biomarkers
- Patient-reported outcome (EQ5D)
- Starting dose of GC
- US scan of the temporal artery including its branches (frontal and parietal) and the axillary artery bilaterally
- Probability score (Additional file 3) GCA Probability score will be calculated on all the patients referred to the FTP to clinically stratify their risk of having GCA and as a measure of severity of the disease

A diagnosis of GCA will be based on revised classification criteria as proposed recently (Dejaco et al. Rheumatology 2016) in the modified GiACTA criteria detailed below. The accuracy of the diagnosis will be evaluated after 6 months.

##### Patients were classified as having GCA if all of the following criteria were met

- Age ≥ 50 years with ESR > 30 mm/hr. or CRP > 10 mg/L
- Unequivocal cranial symptoms of GCA (i.e. new-onset localised headache, scalp or temporal artery tenderness, ischemia-related vision loss, or otherwise unexplained mouth or jaw pain upon mastication) or symptoms of polymyalgia rheumatica (PMR), defined as shoulder and/or hip girdle pain associated with inflammatory morning stiffness
  - *Cranial symptoms defined as new localised head pain, generalised scalp tenderness, tender temporal artery, AION or PION, jaw claudication or tongue claudication in the current study*
  - *PMR symptoms defined as morning stiffness > 1 h with bilateral shoulder pain and/or bilateral hip pain or stiffness in the current study*
- Temporal artery biopsy revealing features of GCA or evidence of GCA by imaging (i.e. ultrasound or cross-sectional imaging such as CTA or PET-CT)

#### Follow-up period: (Additional file 1)

**Fig. 3:**
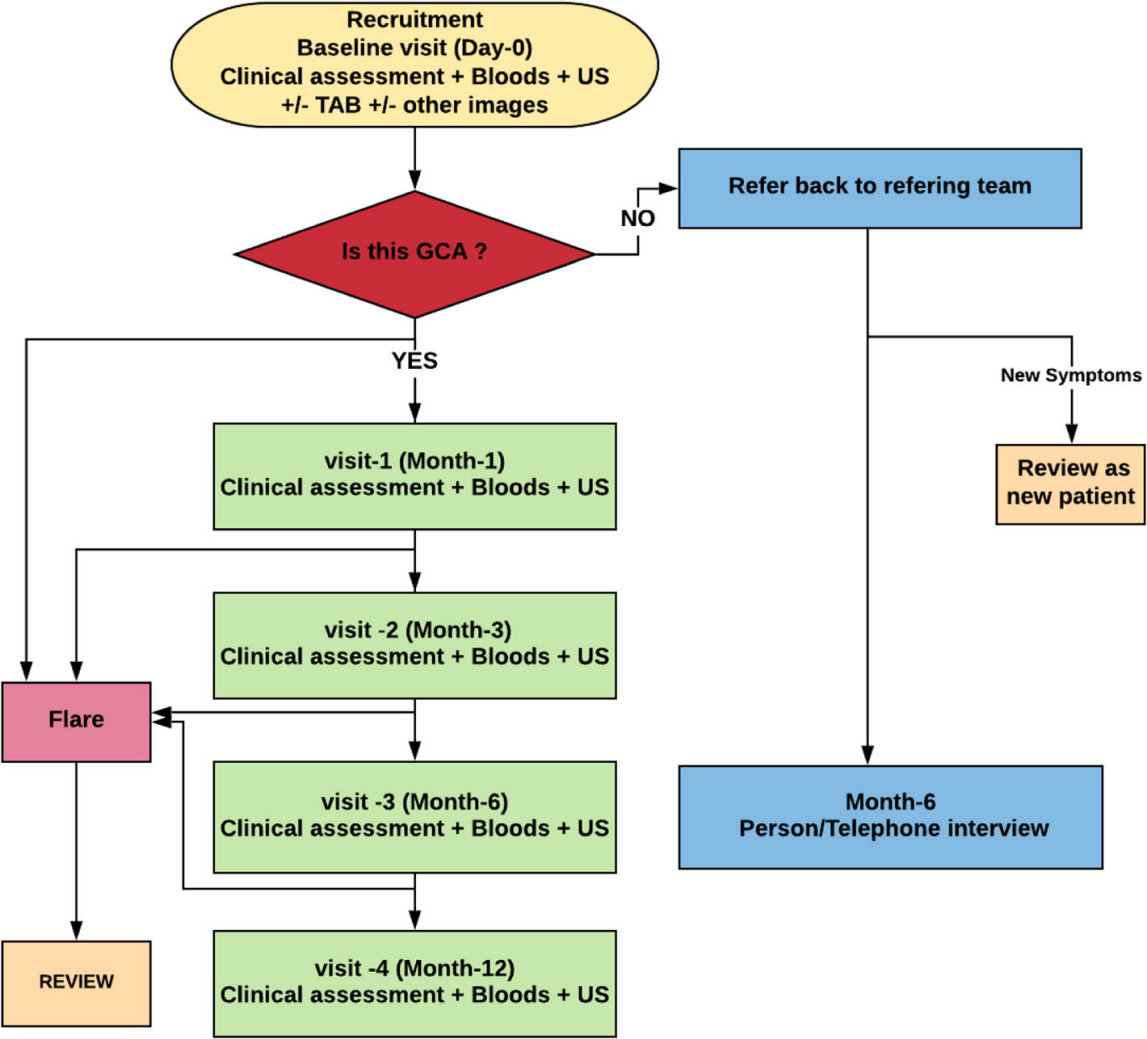
Study Flow chart

Participants who are diagnosed with GCA will be seen for follow-up visits at 1, 3, 6 and 12 months. Participants with a non-GCA diagnosis will be seen or through a telephone interview one further time at 6 months to confirm the non-GCA diagnosis. At any time, point throughout the follow-up period patients may require unscheduled visits if they have symptoms of relapse. Patients will be educated at baseline as to the symptoms that might be expected with relapse and guided to contact their clinician or Rheumatology Research team (if different) immediately (Fig. 3).

For study purposes, relapse means those patients whose GCA symptoms flare or return in response to current standard treatment, that is a tapering regimen of glucocorticoids. Refractory GCA patients are those who do not respond from the outset.

In this phase, following will be assessed:

- Clinical history
- Clinical examination
- Routine bloods including biomarkers
- US scan of the temporal artery including its branches (frontal and parietal) and the axillary artery bilaterally
- Patient-reported outcomes (EQ5D)
- Cumulative GC requirement

**Table:** 

**DEFINITION OF RELAPSE AND REMISSION**	
1. Remission is defined as absence of clinical signs and symptoms of GCA and normalization of ESR [< 30 mm/hr] and CRP [< 10 mg/L]	
2. Relapse is defined as recurrence of symptoms attributable to active GCA, with or without ESR > 30 mm/hr. and CRP > 10 mg/L	
3. The refractory non-remitting disease subjects are those who have had no remission within 6 weeks of initiation of high dose glucocorticoid treatment.	

### Eligibility criteria

Patients with clinical suspicion of GCA referred to the FTC would be eligible for the study subjects to the inclusion and exclusion criteria below.

#### Inclusion criteria

The clinician responsible for the patient’s care will make the diagnosis of GCA as part of the standard of care using the modified GiACTA criteria.

- Age ≥ 50 years with ESR > 30 mm/hr. or CRP > 10 mg/L
- Unequivocal cranial symptoms of GCA (i.e. new-onset localised headache, scalp or temporal artery tenderness, ischemia-related vision loss, or otherwise unexplained mouth or jaw pain upon mastication) or symptoms of polymyalgia rheumatica (PMR), defined as shoulder and/or hip girdle pain associated with inflammatory morning stiffness
  - Cranial symptoms defined as new localised head pain, generalised scalp tenderness, tender temporal artery, AION or PION, jaw claudication or tongue claudication in current study
  - PMR symptoms defined as morning stiffness > 1 h with bilateral shoulder pain and/or bilateral hip pain or stiffness in the current study
•Temporal artery biopsy revealing features of GCA or evidence of GCA by imaging (i.e. ultrasound or cross-sectional imaging such as CTA or PET-CT)
•Participants must have the capacity and willingness to give informed written consent

#### Exclusion criteria

- Participants must not have a previous diagnosis of GCA
- Participants must not have had a previous temporal artery biopsy i.e. as part of diagnostics for previously suspected GCA
- Participants must not be under 18 years
- Participants must not be on treatment with a high dose of steroids (> 7.5 mg) more than 2 weeks prior to the first review in the FTP
- Inability to give informed consent

#### Sampling

The nature of this disease is rare; thus, the number of participants will be collected up to 12 months and will be followed up to 12 months as per the study protocol. Patients will be recruited after referral to the participating site FTP.

##### Participants

The cohort of patients for this study will be recruited from the fast track GCA clinics (FTC), which is currently the standard of care for patients with clinical suspicion of GCA. The FTC has been demonstrated a reduced incidence of vision loss and cost-effectiveness [8]. Patients can be referred to the FTC from General Practitioners (GP), Emergency Department (ED), Ophthalmology or from any other specialities. Initial assessment includes clinical assessment (patient history and physical examination), blood tests (ESR, CRP, full blood count, renal profile, liver function tests), and US of the Temporal and axillary arteries. Those who are diagnosed with GCA will be monitored in the GCA follow-up clinics. Those patients with low probability and a non-GCA diagnosis would be referred back to the primary referral team.

### Intervention

Potential study participants will be identified from patients referred by their GP, ED, Ophthalmology or other specialities to the FTP. For study purposes, referred patients will be informed about the study and provided with a study invitation letter and patient information sheet (PIS) during the first contact with the research team. All participants will need to provide written, informed consent to take part in the study. Due to the nature of the study, researchers will provide as much as information as possible at the time of the first assessment. The research team will answer any questions from the patients. Patients are reassured that their decision will not impact on their standard of care. Those who understand and agreed to participate will be consented and given a unique identification number.

The US of the temporal artery branches and axillary artery on both sides is a key element of the study, which will measure the IMT of each artery and a total halo score (THS) calculated. This score will be used to assess the severity of the disease. It will also be calculated on each follow-up visit to determine how the THS changes with treatment.

### Operator’s experience

- All sonographers participating in this study have experience of scanning more than 30 people with temporal artery and axillary scans and at least 5 cases with GCA.
- All sonographers have completed either face to face or web-based training on the temporal artery and axillary artery scanning requirements for this study.
- All sonographers have completed the online BSR e-learning module on Ultrasound scanning for LVV
- We have documented the experience of sonographers and equipment characteristics with completion of a standardised form (Additional file 4)

### Outcome measures

#### Primary outcome

1. Analysis of data to see how many patients had sustained remission (achieving a daily prednisolone dose of ≤5 mg of glucocorticoid dose equivalent) at 12 months from baseline (one flare is acceptable in this study period). All patients follow the same tapering scheme as outlined in the British Society for Rheumatology (BSR) guidelines (Additional file 2). To then determine if the initial baseline HS correlates with this clinical outcome at 12 months.

#### Secondary outcomes

1. To determine if a change in HS over the 12-month disease monitoring period correlates to prognosis
2. To determine if there is any correlation of HS to quality of life measures, as assessed by EQ5D
3. To determine any correlation between the HS and biomarkers of GCA patients
4. Evaluate if the Probability Score (Additional file 3) prospectively correlates with GCA outcomes at 12 months
5. To determine the diagnostic accuracy of the HS for discriminating GCA from non-GCA Reference standard for the diagnosis of GCA will be the clinical diagnosis after 6 months follow-up.
6. To determine the diagnostic accuracy of the GCA probability score for discriminating GCA from non-GCA patients. The reference standard for the diagnosis of GCA will be the clinical diagnosis after 6 months follow-up.

### Data analysis and monitoring

Descriptive statistics such as mean (with standard deviation), median (with range) and percentages will be used for reporting HS, relative change in HS, number of patients in remission with prednisolone dose ≤5 mg daily after 12 months, cumulative prednisolone dose at 12 months follow-up, time to first relapse, number of relapses, levels of inflammatory markers, quality of life questionnaire outcomes and GCA probability scores. Temporal artery, axillary artery halo scores and the total halo score (temporal score plus axillary score) will be calculated.

### Primary outcome analysis and power calculation

Percentages of GCA patients in remission with a prednisolone dose of ≤5 mg per day will be determined at 12 months follow-up. A ROC analysis of baseline HS will be performed to identify the optimal HS cut-off point that discriminates between patients reaching remission and those that do not. Subsequently, the Chi-square test will be used to compare remission rates at 12 months follow-up between patients with a HS above the optimal cut-off point versus those with a HS below the optimal cut-off points.

A power calculation was performed to determine the number of patients needed for investigating this primary outcome. Based on two previous studies, it is expected that 45% of GCA patients will be in remission at 12 months with a prednisolone dose of ≤5 mg per day [40, 41]. For the current study, we propose that a 40% difference in patients reaching sustained remission at 12 months follow-up is clinically relevant.

As the optimal prognostic HS cut-off point is not yet known, we propose that a 25% versus 75% distribution is still clinically relevant. If the smallest group becomes smaller (and the biggest group bigger), we believe risk stratification by HS would have limited overall value for clinical practice. With an alpha of 0.05 and power of 0.80, we calculate that 61 GCA patients are needed for the study.

Taken into consideration a 10% loss of patients during 12 months follow-up, we expect that 68 GCA patients should be initially recruited into the study.

In our experience, 25% of patients entering a GCA FTP, will be ultimately diagnosed with GCA after 6 months follow-up. Thus, we anticipate that we would need to recruit a total of 272 patients suspected of having GCA in our study, of which 68 are eventually diagnosed as having GCA.

G.Power 3.1.9.4

**Table:** 

**z tests**	Proportions: Difference between two independent proportions	
**Analysis**	A priori: Compute required sample size	
**Input**	Tail(s)	= Two
	Proportion p2	= 0.65
	Proportion p1	= 0.25
	α err prob	= 0.05
	Power (1-β err prob)	= 0.80
	Allocation ratio N2/N1	= 3
**Output**	Critical z	= 1.9599640
	Sample size group 1	= 15
	Sample size group 2	= 46
	Total sample size	= 61
	Actual power	= 0.7979079

### Secondary outcome analysis

- In patients with a clinical diagnosis of GCA: the prognostic value of the absolute and relative change in HS between baseline and 1 month’s follow-up will be investigated in a similar analysis as mentioned under the primary outcome analysis
- In patients with a clinical diagnosis of GCA: we will perform a paired analysis of the HS measured at different time points by paired t-test or Wilcoxon signed-rank rest depending on normality of the data
- In patients with a clinical diagnosis of GCA: correlation between HS and measures of quality of life will be determined by Pearson or Spearman’s rank correlation coefficient depending on normality of data
- In patients with a clinical diagnosis of GCA: correlation between HS and inflammatory markers in blood will be determined by Pearson or Spearman’s rank correlation coefficient depending on normality of data
- In patients with a clinical diagnosis of GCA: the prognostic value of the GCA probability score will be assessed similar to the analysis of the prognostic value of the HS as mentioned under the primary outcome analysis
- In all patients suspected of having GCA: the diagnostic accuracy of the HS for discriminating GCA from non-GCA patients will be determined by ROC analysis and the Youden index. Sensitivity, specificity and likelihood ratios at the optimal diagnostic cut-off point will be evaluated. The reference standard for the diagnosis of GCA will be the clinical diagnosis after 6 months follow-up.
- In patients with a clinical diagnosis of GCA: the diagnostic accuracy of the HS for discriminating relapsing and non-relapsing GCA patients during follow-up measurements. Sensitivity, specificity and likelihood ratios at the optimal diagnostic cut-off point will be evaluated. Relapse definition is described elsewhere.
- In patients with a clinical diagnosis of GCA: the predictive effect of the baseline HS and GCA probability score on GCA patients achieving remission at 12 months with prednisolone dose of ≤5 mg will be evaluated by multivariate logistics regression analysis.
- In patients with a clinical diagnosis of GCA: the predictive effect of the HS and GCA probability score on cumulative prednisolone dose at 12 months follow-up will be evaluated by multivariate linear regression analysis.
- In addition to the total halo score in the axillary and temporal artery, changes in individual vessel halo grades will be analysed.

## Discussion

US is a non-invasive procedure, safe and easily accessible and repeatable in the clinic setting, without any radiation exposure to the patient or the sonographer. EULAR recommends US as a first choice imaging investigation in suspected GCA [27], and BSR strongly recommends US or TAB as a confirmatory test in suspected GCA [13]. A recent study *Monti* et al. suggests the use of US as a surrogate tool to replace TAB [42]. US has now become an essential part of the workup of GCA in many centres. However, current US practice in GCA is to declare the test either positive or negative for the ‘Halo sign’ in a dichotomous manner. The extent and severity of the halo sign in assessing disease diagnosis (in the context of differing pre-test probabilities), severity and prognosis is yet to be studied.

Halo is a dark hypoechoic area around the vessel lumen representing the vessel wall inflammation. In temporal arteries, halo compression sign, with a video in the transverse plane will be assessed to confirm all diagnoses of GCA. Non-compressible halo is the key lesion in GCA. Halo thickness will be measured in TA, its branches and axillary arteries. We will be using the published cut off values of the IMT of TA as assessed by the high-frequency probe (22 MHz) [37]. We will use the 18 MHz probe in this study which is compliant with EULAR recommendations of using a probe of frequency > 15 MHz. We recently developed a halo score grading the halo thickness in temporal and axillary arteries [39]. Ultrasound halo score correlates with the vascular inflammation in GCA and strongly associated with ocular ischemia in GCA [39].

Although, the US is a useful tool to study the haemodynamic and morphology of the blood vessels [43] it remains a challenge to interpret of the morphological changes in the different size of the blood vessels. A follow-up study observed an 85% reduction of the vessel wall in temporal artery with treatment contrasted to the large vessels showing a reduction of 45% [44]. A possible explanation for this is the inclusion of popliteal and femoral arteries which are frequently involved by atherosclerosis. Our current study may have a potential solution to this as it will validate the cut off scores for each temporal artery branch and the axillary arteries and sum these changes by measuring the total halo score.

The identification of prognostic factors in GCA is both timely and needed. Recent BSR guidelines recommend initiating high dose of GC immediately in highly suspected patients with GCA [13]. At presentation, extensive vascular involvement of both cranial and large vessels evidenced by US showed a poor response to GC treatment in GCA and often required steroid-sparing agents [45]. A case series showed a significant vessel wall reduction evidenced in US and PET CT in response to Tocilizumab treatment in large vessel GCA [46]. The GiACTA trial has shown that IL-6R blocking therapy may help to sustain glucocorticoid free remission. A prognostic marker, such as the HS, could help to stratify GCA patients for an appropriate treatment regimen. IL-6R blockade switches off the inflammatory marker response, making it difficult to use traditional biomarkers such as CRP to measure disease activity. Therefore, an independent HS, and changes in that score during treatment and follow-up, may be a more objective measure of response, rather than relying only on patient reported symptoms, clinical assessment and acute phase markers (CRP).

## Supplementary information

**Additional file 1.** Study schedule.

**Additional file 2.** Prednisolone tapering (BSR guidelines).

**Additional file 3.** Probability Score.

**Additional file 4.** Sonographer pro-forma.

## Data Availability

Research team members will ensure participants’ anonymity is maintained. Participants will be identified by a unique study number on all documents and any electronic database. All documents will be stored securely and only accessible by research team members and authorised personnel. The data will be saved for a minimum of 5 years. The study will comply with the General Data Protection Regulation (GDPR), which requires data to be anonymised as soon as it is practical to do so. Chief investigator and the research team members have access to the full dataset, which is electronically saved in an encrypted file. The study will allow site investigators to access the full dataset if the steering group approves formal request with their plans. The collected data will be stored electronically, and the consent forms will be securely store in a storage facility. The chief investigator is responsible for all the data stored securely. On completion of the study, the study data will be published in a medical journal.

## Abbreviations

BSR: British Society for Rheumatology
DMARD: Disease modifying anti rheumatic drug
FTP: Fast Track Pathway
GC: Glucocorticoids
GCA: Giant Cell Arteritis
HS: Halo Score
PMR: Polymyalgia Rheumatica
TAB: Temporal Artery Biopsy
THS: Total Halo Score
US: Ultrasound
