# Supplementary material for: Halo score (temporal artery, its branches and axillary artery) as a diagnostic, prognostic and disease monitoring tool for Giant Cell Arteritis (GCA)
Source: BMC Rheumatol. 2020 Aug 18;4:35. doi: 10.1186/s41927-020-00136-5 (PMC7433165; doi:10.1186/s41927-020-00136-5)
Supplement: Supplementary file 1 — Additional file 1. Study schedule. [file 41927_2020_136_MOESM1_ESM.docx]

**APPENDIX 1**

**Study Schedule for GCA Patients**

| **Visit** | **Baseline** | **Week 4** | **Week 12** | **Week 24** | **Week 52** |
| --- | --- | --- | --- | --- | --- |
| Confirmation of patient consent | X | X | X | X | X |
| Observations (HR, RR, BP, Temp.) | X | X | X | X | X |
| Clinical assessment (History and examination) * | X | X | X | X | X |
| Medication review | X | X | X | X | X |
| Cumulative GC dose calculation | X | X | X | X | X |
| Probability score | X |  |  |  |  |
| Vascular ultrasound and halo score | X | X | X | X | X |
| Study bloods** | X | X | X | X | X |
| Lipid profile | X |  |  | X | X |
| HbA1C | X |  |  | X | X |
| Urinalysis | X | X | X | X | X |
| EQ5D | X |  | X | X | X |

*Including GC related AE

** FBC, Renal profile, Liver function tests (including AST), ESR, CRP

HR – heart rate, RR – Respiratory rate, BP – Blood pressure, GC - Glucocorticoid
