# Supplementary material for: Halo score (temporal artery, its branches and axillary artery) as a diagnostic, prognostic and disease monitoring tool for Giant Cell Arteritis (GCA)
Source: BMC Rheumatol. 2020 Aug 18;4:35. doi: 10.1186/s41927-020-00136-5 (PMC7433165; doi:10.1186/s41927-020-00136-5)
Supplement: Supplementary file 2 — Additional file 2. Prednisolone tapering (BSR guidelines). [file 41927_2020_136_MOESM2_ESM.docx]

**APPENDIX 2**

**PREDNISOLONE TAPERING**

*BSR guideline*

*"40–60 mg prednisolone (not less than 0.75 mg/kg) continued for four weeks (or until resolution of*

*symptoms and lab abnormalities):*

*• then dose is reduced by 10 mg every two weeks to 20 mg*

*• then by 2.5 mg every two to four weeks to 10 mg*

*• then by 1 mg every one to two months, provided there is no relapse."*

*BSR guideline allows different tapering speed in GCA patients. For prognostic study it is important that patients are tapered according to a fixed protocol. It would be most straightforward if there was one exact protocol for all patients. But if some variation is wanted, please state:*

*- when patients are receiving methylprednisolone 500 mg or 1000 mg IV*

*- when patients are started on 60 mg prednisolone or 40 mg prednisolone*

*- when patients are decreasing steroids by 2.5 mg every 2 weeks or 4 weeks*

*- when patients are decreasing steroids by 1 mg every one month or 2 months*
