# Supplementary material for: Halo score (temporal artery, its branches and axillary artery) as a diagnostic, prognostic and disease monitoring tool for Giant Cell Arteritis (GCA)
Source: BMC Rheumatol. 2020 Aug 18;4:35. doi: 10.1186/s41927-020-00136-5 (PMC7433165; doi:10.1186/s41927-020-00136-5)
Supplement: Supplementary file 3 — Additional file 3. Probability Score. [file 41927_2020_136_MOESM3_ESM.docx]

**APPENDIX 3 :** Probability Score

| WEIGHTAGE | -3 | 0 | +1 | +2 | +3 |
| --- | --- | --- | --- | --- | --- |
| Age |  |  | 50-60 | 60 – 65 | >65 |
| Sex |  |  | M | F |  |
| Onset |  | >24 wks | >12  wks | 6-12 wks | < 6 wks |
| Inflammatory marker:  CRP |  | 0-5 | 6-10 | >10 | >25 |
| SYMPTOMS: |  |  |  |  |  |
| Cranial - Head and scalp pain |  | N | Y |  |  |
| Constitutional |  | N | Single |  | Combination |
| Polymyalgia |  | N |  | Y |  |
| ischemic (uniocular, Blurring, diplopia, amaurosis, jaw/tongue pain |  | N |  |  | *Y* |
| SIGNS: |  |  |  |  |  |
| Visual (AION/CRAO/Field/RAPD) |  | N |  |  | *Y* |
| Temporal Arterial abnormality |  | N | Tenderness | Thickening | Loss of pulse |
| Extra-cranial abnormalities |  | N | Thickening | Bruit | Loss of pulse |
| Cranial N palsy (3,4,6) |  | N |  |  | Y |
| Alternative diagnosis as or more likely than GCA |  |  |  |  |  |
| Active infection | Y | N |  |  |  |
| Active cancer | Y | N |  |  |  |
| Other systemic rheumatological diseases | Y | N |  |  |  |
| Other head and neck pathology | Y | N |  |  |  |
| TOTAL SCORE |  |  |  |  |  |

N.B. Negative scoring can only be used once, if more than one negative feature applies the maximum would still be -3.
