# Supplementary material for: Halo score (temporal artery, its branches and axillary artery) as a diagnostic, prognostic and disease monitoring tool for Giant Cell Arteritis (GCA)
Source: BMC Rheumatol. 2020 Aug 18;4:35. doi: 10.1186/s41927-020-00136-5 (PMC7433165; doi:10.1186/s41927-020-00136-5)
Supplement: Supplementary file 4 — Additional file 4. Sonographer pro-forma. [file 41927_2020_136_MOESM4_ESM.doc]

**APPENDIX 4**

**HAS GCA - Pre-Study Sonographer Experience Data Collection Pro-Forma**

|  | **Response** |
| --- | --- |
| **Surname** |  |
| **Forename** |  |
| **Grade (e.g. Consultant, SpR, Fellow, Sonographer)** |  |
| **Site (e.g. Hospital)** |  |
| **Provision for assessment of acute GCA at your site (Please provide details including any dedicated services e.g. Fast Track Pathway)** |  |
| **Approximate number of patients seen with suspected new-onset GCA at your site in 1 calendar year** |  |
| **Personal scanning experience (years)** |  |
| **Evidence of formal training (Please detail any previous courses, training programmes or placements)** |  |
| **Number of temporal artery US scans performed** | **Last year:** |
|  | **Total:** |
| **Number of acute GCA cases scanned (‘Hot Scans’)** | **Last year:** |
|  | **Total:** |
| **Details of US machine ( make, model, probe, frequency)** |  |
| **Signature and Date** |  |
